# Formative research on the feasibility of hygiene interventions for influenza control in UK primary schools

**DOI:** 10.1186/1471-2458-9-390

**Published:** 2009-10-15

**Authors:** Wolf-Peter Schmidt, Catherine Wloch, Adam Biran, Val Curtis, Punam Mangtani

**Affiliations:** 1 Department of Infectious and Tropical Diseases, London School of Hygiene and Tropical Medicine, UK

## Abstract

**Background:**

Interventions to increase hand washing in schools have been advocated as a means to reduce the transmission of pandemic influenza and other infections. However, the feasibility and acceptability of effective school-based hygiene interventions is not clear.

**Methods:**

A pilot study in four primary schools in East London was conducted to establish the current need for enhanced hand hygiene interventions, identify barriers to their implementation and to test their acceptability and feasibility. The pilot study included key informant interviews with teachers and school nurses, interviews, group discussions and essay questions with the children, and testing of organised classroom hand hygiene activities.

**Results:**

In all schools, basic issues of personal hygiene were taught especially in the younger age groups. However, we identified many barriers to implementing intensive hygiene interventions, in particular time constraints and competing health issues. Teachers' motivation to teach hygiene and enforce hygienic behaviour was primarily educational rather than immediate infection control. Children of all age groups had good knowledge of hygiene practices and germ transmission.

**Conclusion:**

The pilot study showed that intensive hand hygiene interventions are feasible and acceptable but only temporarily during a period of a particular health threat such as an influenza pandemic, and only if rinse-free hand sanitisers are used. However, in many settings there may be logistical issues in providing all schools with an adequate supply. In the absence of evidence on effectiveness, the scope for enhanced hygiene interventions in schools in high income countries aiming at infection control appears to be limited in the absence of a severe public health threat.

## Background

Although generally not having the highest mortality, school children bear a substantial burden of influenza-related morbidity and other infections [1,2]. Clinical attack rates in children in the current H1N1 influenza A epidemic may be twice that of adults [3]. Schools and school children have been shown to play a major role in the spread of influenza during epidemics [4,5]. As a component of the layered interventions for mitigating a pandemic (that may include vaccination, prophylactic use of antiviral drugs and school closure) school-based interventions targeting hand hygiene behaviour in school children could be important. Hand hygiene interventions in schools could be of low cost and, apart from any direct effect on influenza, may reduce other infections and sickness related absence in pupils and teachers [6].

However, it is unclear how appropriate and feasible improving hand hygiene in schools is. This needs to be established to facilitate compliance. The recent spread of novel swine-like H1N1 influenza in schools in the UK has raised interest in improved hand hygiene. We conducted formative research in four primary schools in London to establish the need for enhanced hand hygiene interventions, to identify barriers to their implementation and to test their acceptability and feasibility.

## Methods

### Selection of schools

The four schools included in the research were sampled purposively from a large borough in East London with great ethnic and socioeconomic diversity, aiming to give a range of ethnic and socioeconomic backgrounds: The sample included two infant schools (reception year till year 2), one primary school (years 1-6) and one junior school (years 3-6). Children in these schools were from various ethnic backgrounds (mainly White British, South Asian, West African, Arabic, Eastern European). The proportion of pupils receiving free school meals (an indicator of socio-economic condition) ranged between 8% and 30%.

We selected one class per school for inclusion in the formative research resulting in four classes overall. The selected class grades included: year 1 (one class), year 2 (two classes), year 6 (one class in junior school).

The choice of the classes was left to the head teachers, who were asked to choose a "typical" class led by a class teacher with an average level of teaching experience. Head teachers appeared to avoid classes where low discipline was expected. Random sampling was not accepted by the schools.

### Components of the pilot study

Formative research serves to answer key questions prior to the design of a behaviour change intervention. Typically these include: What precise behaviour should be targeted? Who are the key target audience(s)? What are the motivators for and barriers to changes in behaviour? And how best to reach target audiences [7-10]? We employed a variety of qualitative research methods that we used previously to study personal hygiene in school children [11,12]. The components of the research were: Key informant interviews (head teachers, teachers, school nurses), semi-structured interviews, artwork, group discussions and semi-structured essay questions (pupils). We conducted pilot trials during which teachers were given the task of testing different ways to conduct organised classroom hand hygiene over a period of time.

The key informants' interviews included questions regarding current activities, perceived importance of hygiene activities for children in relation to other educational activities, motivations for implementing hygiene activities, current capacity to implement activities, perceived barriers and constraints to implementing them. Semi-structured interviews, essay questions and group discussions with children included questions on illness perception, on how people catch influenza and colds, and hygiene behaviour. The younger children were asked to draw a picture of an occasion when they thought that someone should have washed their hands but didn't. The children then explained their drawing to the researcher. Perception of the symptoms of colds and flu was also explored using a game whereby the children were given cards with 4 different illnesses (chicken pox, hay fever, cold and flu). They were then given cards depicting a variety of symptoms and asked to match the symptoms to the illnesses. Cartoon illustrations were added to the cards to help any younger children with limited literacy.

Older children were asked to write a short paragraph and draw pictures of how they thought germs were passed from one person to another. Semi-structured interviews were conducted with groups of 2-3 children so that the children did not feel intimidated. All question guides were pilot tested in a small group of children.

We also tested staff and childrens' acceptability of three different hand hygiene products for organised hand hygiene in the classroom: liquid soap, alcohol-based hand sanitiser (liquid) and alcohol-based hand sanitiser (gel). We measured the time it takes for the whole class to practice hand hygiene. These behaviour trials were done twice for each hand hygiene product in the presence of the researcher, and then for 2 days (4 times per day) by the class under supervision of the teacher using the product preferred by the class (hand gel in all cases). The behaviour trials were followed by interviews and group discussions with pupils and teachers about the different approaches. During the behaviour trials, no specific hygiene education was offered to the classes. However, in all cases the teachers explained to the children why such exercise might be important for health.

Interviews with children were tape recorded and transcribed. Interviews with teachers and nurses were not tape recorded. The interviewer made notes of key points arising and verbatim quotes. We performed manual thematic content analysis of transcripts and interview notes in order to provide a descriptive account of the ways in which respondents perceived cold and flu symptoms and transmission, salient reasons for hand washing and common barriers to hand washing. Topics were decided in advance and covered symptoms of colds and flu, transmission of colds and flu, reasons/occasions for washing hands, knowledge and attitudes about hygiene and barriers in relation to hand washing. Thematic grouping was according to themes developed by the authors through reviewing the data.

The study was approved by the ethics committees of the London School of Hygiene and Tropical Medicine and the local NHS primary care trust and conducted between autumn 2006 and spring 2007. Informed written consent was obtained from all parents of children taking part in research activities that were not on class-level (interviews, artwork, semi-structured essay questions). All parents of the classes involved received an information sheet offering to opt out of class-room activities (behaviour trials, teacher-led discussions). None of the parents chose to opt out.

## Results

### Disease concepts of children

Children described colds and flu in terms of their experiences of symptoms. Having a cold meant: '*A cough, sneezing very too much*.' (Year 1 child), '...*having a blocked nose*...' (Year 1 child), '...*when you go to sleep you can't breathe*' (year 6 child). Flu was described in similar terms but as a more unpleasant or persistent condition. '...*100 times worse than a cold*'(Year 6 child). '...*sneezing and keep on sneezing*.' (Year 1 child). '*They would be really sick and cry*' (Year 1 child). Symptoms of fever were also associated with flu. '...*you have a temperature*...' (year 6 child). '*They get a temper thing*...' (Year 1 child).

Even children as young as Year 1 showed awareness of person to person transmission and the idea that germs transmit infection. '*When you have a sneeze your germs will go to other people and other people will have sneezes as well*' (Year 1 child). '*If I touch my baby sister she will have the germs*' (Year 1 child). Children generally attributed the transmission of germs to coughing and sneezing, touching or being in proximity to an infected person. '*If they sneeze or cough the germs get to you*' (year 6 child). 'When other people have got it and you go near them then you will get it' (Year 6 child). Some also discussed catching a cold after being cold, '*They don't wear a coat and they go outside...in the Winter*' (Year 6 child). The short essays produced by Year 6 children reiterated these routes of transmission but also mentioned not washing hands after the toilet or sneezing, and sharing drinks. Illustrative quotes are given in Table 1. As one head teacher said '*They understand about washing hands and germs*.'

**Table 1:** Children's explanations of illness transmission, and reasons for practicing hand washing and respiratory hygiene

**Topic**	**Theme**	**Typical quotes**
Transmission of flu and colds	Germs	'When you have a sneeze your germs will go to other people and other people will have sneezes as well' (Year 1)
		'If I touch my baby sister she will have the germs' (Year 1)
		'If they sneeze or cough and the germs get to you' (Year 6)
	Person to person	Someone in my family had it, then I got it too' (Year 2)
		'It goes from one person to another' (Year 2)
		'If someone is coughing or sneezing around them then they will catch it' (Year 6)
	Proximity/contact	'When you touch people who have flu or other diseases' (Year 6)
		'Because they stand next to each other' (Year 1)
		When other people have got it and you go near them then you get it. (Year 6)
		'If you go close to someone with flu' (Year 6)
	Cold	'It's cold and you didn't wear a coat, then you make cold' (Year 1)
		'They don't wear a coat when they go outside, mostly in Winter'' (Year 6)
	Dirt/contamination on hands	'If they don't wash their hands after they go to the bathroom' (Year 6)
		'Eating without washing our hands' (Year 6)
		'If you touch something dirty and then touch your face' (Year 6)
	Other	'Some immune systems can't deal with it' (Year 6)
		'...when you are not active' (Year 6)

Occasions for washing hands	Eating	'to eat our food' (Year 1)
		'having dinner or tea' (Year 1)
		Before eating (Year 6)
		'Before I eat I have to wash my hands' (Year 6)
		'Before eating' (Year 6)
	Toilet	'After toilet' (Year 6)
		'After toilet' (Year 1 picture)
	Visible or sensory dirt	'...if you've played in the garden and touched soil' (Year 6)
	Contact with contaminated object	'If they do something dirty in the garden' (Year 1)
		'...after eating with hands' (Year 6)
	Sneezing	'After touching a bin' (Year 6 picture)
		'After sneezing on hands' (Year 6 picture)

Reasons for hand washing	Avoid germs	'Because you might get germs off the toilet' (Year 1)
		'I went to the museum and they said germs go on your hands every 5 seconds.' (Year 6)
		'Hygiene, you always have germs on your hands so when you eat without washing your hands all those germs go into your body.' (Year 6)
		So you don't get germs on your mouth, face and hands (Year 1)
		'Because if you don't you will get germs and you will start to be ill' (Year 1)
		To get rid of germs (Year 6)
	Avoid illness	'So I don't get ill' (Year 2)
	Remove visible dirt	'Cleanliness, so there's no bits on your hands and you're not muddy or dirty or anything.' (Year 6)
		'Because when you do dirty stuff like handstands you might get your hands dirty.' (Year 1)

Reasons for not washing hands	Don't want to/like dirt	'Because they don't want to, don't feel like it' (Year 1)
		'They like their hands to be dirty' (Year 1)
	No need	'They're not really bothered and they don't really care.' (Year 6)
	Competing priorities	Because they don't eat'
		'Because their favourite programme is coming and they don't want to miss it' (Year 1)
	Ignorance	'...playing a game and not want to miss anything' (Year 6)
		'They don't know the reasons why they should' (Year 6)
	Poverty/moral judgement	'Because they are poor and they stink' (Year 1)

Respiratory hygiene practices	Turn away	'Face the other way so no one is there' (Year 1)
		'Turn my head away from everyone' (Year 6)
	Use Tissue	'Use a tissue' (year 6)
	Cover with hands	'Cover my nose...get a tissue and put it' in the bin (Year 1)
		'You need lots of tissues for sneezing so you can stop the sneeze go anywhere' (Year 1)
	Cover and wash hands	'Put your hands up in front' (Year 1)
		'You put your hand in front of your mouth' (Year 1)
		'Use a tissue or your hand and wash your hand afterwards' (Year 6)
		'Sneeze in hands and wash hands' (Year 6)
		'Cover my mouth and wash hands after' (Year 6)
		'If you cover your mouth with your hands when you sneeze it would be even better if you wash hands afterwards' (Year 6)

Reasons for respiratory hygiene	Protect others	'So they don't get germs on their face' (Year 1)
		'When you sneeze the germs fo out of your nose and onto other people' (Year 1)
		'Sneeze on your little sister and she won't go to nursery school'
		'It protects other people from catching your germs' (Year 6)
	Stop germs	'Use a tissue so germs go on the tissue and don't spread' (Year 6).
		'When you sneeze the germs travel along to another person but a tissue blocks the germs' (Year 6)
		'Germs get caught in the tissue and don't spread everywhere' (Year 6)
		'The tissue traps germs...and they can't get out' (Year 6)

### Children's knowledge and attitudes towards hand and respiratory hygiene

Virtually all children said that their parents instructed them to wash their hands, but older siblings and wider family members were also mentioned. Year 6 children frequently mentioned that their own common sense prompted them to wash their hands. School teachers and other adults were only mentioned after probing further. Reasons for hand washing stated by children were to remove dirt and germs, to prevent germs going into their mouths when eating and to prevent illness. '...*if you don't [wash hands] you will get germs and ...start to be ill*' (Year 6 child). Illustrative quotes are given in Table 1. Hand washing after visiting the toilet was infrequently mentioned, and mostly only after probing. Most children, especially those with an Asian or Middle-Eastern background, thought that hand washing was most important before eating and reported this as the occasion when their parents most frequently told them to wash their hands.

The need to use a tissue and turn away from other persons when coughing and sneezing was common knowledge among the children regardless of age (although observation of hygiene behaviour in the classroom revealed that this was rarely practiced). Again children claimed that they did this because coughing and sneezing were major routes of germ transmission. ' [people tell you to sneeze into a tissue] ...*so they don't get germs on their face*.' (Year 1 child). '*When you sneeze germs travel along to another person but a tissue blocks the germs*...' (Year 6 child). *'Use a tissue so germs go onto the tissue and don't spread' *(Year 6). Illustrative quotes are given in Table 1.

The Year 1 and 2 children suggested that some children don't wash their hands because they think their hands are clean, because they are rushing so they don't miss out on something, or because some children like dirt (Table 1). Year 6 children thought that ignorance, lack of care and laziness were reasons why some children don't wash their hands, as well as rushing to return to something more interesting. The pictures drawn by infant children of occasions when people should have washed their hands but didn't depicted people touching dirt or dustbins, as well as going to the toilet or sneezing without washing their hands.

### Key informant interviews with head teachers and teachers

We interviewed four head teachers and six class teachers. The views of these two groups differed little in most aspects and are therefore described together, unless stated otherwise. Hygiene education (e.g. hand washing, using a tissue when sneezing, not eating with hands, washing fruits before consumption) is formally taught as part of general health education in all of the schools, sometimes aided by a school nurse. Hygiene education is also integrated in the daily life at the schools. Teachers say they observe the hygiene behaviour of children closely and encourage or enforce personal hygiene, e.g. hand-washing after sneezing, going to the toilet or contamination with dirt. In most classes they stated that children all wash hands in the classroom before the lunch break under the supervision of the teacher. Although as one teacher said *'The children wash their hands before lining up for lunch but it is often a quick run under the tap.'*

Some teachers thought there could be more formal teaching on hygiene, but also stressed that it competes with other aspects of health education, such as healthy eating, drugs and sex education, especially for the older children. Hygiene was rated as less important than these issues, but does, however, have a prominent role in the first school years. Nearly all teachers thought that the current activities were adequate, given the time constraints. There was also scepticism with regard to implementing more formal hygiene interventions. One head teacher said: '*No more state interventions! Hygiene is about applying common sense*.' However, most teachers thought that during a major public health threat like an influenza pandemic there would be sufficient opportunity to temporarily enforce much more intensive hygiene measures.

Teachers reported several motivations for teaching and enforcing good hygiene behaviour in children. Some found it simply disgusting to watch children not behaving hygienically, others felt it put both the teacher and other children at risk for infection. *'I don't want to get your germs' *is how one teacher put it. The commonest motive to teach and encourage hygiene practices was that teachers felt it is important for the development of the children. Children thereby learn to take care of themselves and others. One teacher said: '*Knowledge *[about the importance of Hygiene] *makes children more aware of other people around them*.' Teachers also recognised the potential importance of the home environment in influencing hygiene behaviours. *'Hygiene is not under the children's control at home, it's down to the parents' *said one teacher. Another thought it *'interesting to know to what extent children take their acquired hygiene behaviour home and practice it there as well*.' One teacher was more general: *'It's the well-being of the children. A good grounding in younger years of basic ideals stands them in good stead for later years*.' These quotes suggest that teachers regard hygiene as part of their educational remit. Infection control as a public health measure was not specifically mentioned, although several teachers and head teachers with experience in teaching at socio-economically less well-off schools or schools with high absenteeism hoped that hygiene (in particular hand-washing) would reduce absenteeism.

### Key informant interviews with school nurses

We interviewed 3 school nurses. The interviews revealed that infection control forms only a minor part of their work. Most of their time is spent on child protection issues, giving advice to parents, managing school children with special needs and other tasks. They are interested in contributing more to disease prevention and infection control including hygiene interventions, but their current workload does not allow many activities. Teachers were in a better position to implement and supervise hygiene practices in schools, although they saw a role for school nurses to reiterate messages about germs and hygiene, e.g. when there are special events at the school dedicated to health education. Such events (for example "health week") are used by school nurses for health education including hygiene.

In contrast to teachers, the main motivation of school nurses promoting hand hygiene in schools appeared to be infection control. They see a role for school nurses in implementing infection control measures in schools during an influenza pandemic. They thought that schools as well as school and health authorities would expect them to deliver such measures. The school nurses felt a need for more practical pandemic guidelines, for example on how many times hand hygiene should be practiced in schools and what products to use. Specific guidelines may help to build trust between school, parents and school nurses during a pandemic.

School nurses said that children found hygiene teaching great fun. For example, they enjoyed a demonstration of hand-washing using UV light to highlight the importance of soap. They also thought children were impressed with TV commercials, especially a particularly disgusting one on sneezing and coughing and hand washing that almost all the children seemed to have seen.

### Behaviour trials

Organised classroom hand hygiene with liquid soap was far more time consuming (on average 8 minutes for 30 children with little variation) than the alcohol rubs (on average 3 minutes) even if conducted in a rushed and superficial manner. One teacher commented on the advantage of the alcohol rubs *'They didn't need to queue up at the sink, with hand sanitizer you give a squirt and they go back to task.' *The demands of teaching also competed with organised hand washing. As one teacher reported *'We forgot sometimes when we were involved in teaching.'*

The reactions of the children to using soap or alcohol gel are shown in Table 2. The rinse-free alcohol gel was generally received well by children and teachers alike. Two children out of approximately 90 children complained about a mild skin rash on the hand which appeared at the site of a fresh scratch after the gel was applied. The liquid alcohol-based sanitiser was regarded as much less suitable by teachers and children because of its strong smell and the fact that it dripped on the ground.

**Table 2:** Attitudes of children towards classroom hand hygiene

	**Hand washing with soap and water**	**Gel-based alcohol hand sanitiser**
Motivations	'smells nice'	'quicker than soap'
	'hands clean and nice'	'easier than soap'
	'germs are washed away'	'don't need sink'
	'you can see soap all over hand'	'I like the smell'
		'The doctor's gel felt fresher on your hands'

Barriers	'too many children - making classroom wet'	'too smelly'
	'too much shoving and pushing'	'hands felt funny'
	'takes too long'	'had a scratch and then it stings'
	'too many people dripping soap over everyone while waiting'	'Pen ink doesn't come off with gel.'
	'too much rush'	' [I liked the] Doctor's gel but I didn't like the smell'
	'didn't like to use wet towel'	

Conventional hand washing with soap and water was found feasible but very disruptive. Children complained that the long queue in front of the sink left no time for thorough hand washing. With the rinse-free alcohol rubs there was no need to queue - the teacher could go around with the gel bottle and administer it to every child into their hands. This gave children more time to rub their hands. Two teachers were concerned that using rinse-free hand sanitisers could interfere with the teaching of hand washing with soap as practiced in everyday life. The teachers wanted children to understand that hand washing with soap is still the best method since hand sanitisers may be rarely available, especially at home. The teacher felt that it should be made clear that hand sanitisers are only for situations where one cannot use conventional soap and water. On the whole, behaviour trials were well received by children and teachers. Many children engaged enthusiastically in the activities.

## Discussion

This formative research study explored the factors that could be important for the implementation of hygiene promotion during an influenza pandemic.

The study demonstrated that teachers were already making some efforts to encourage hygiene in class. The reasons included inculcating good manners in children, avoiding behaviour that appeared disgusting, avoiding absenteeism, and protecting themselves and others from infection. Teachers were able to implement intensive hand hygiene measures when asked to do so. Using hand gels appeared to be the most feasible approach to organised classroom hand hygiene. However, teachers in general did not see teaching hygiene as a particular priority compared to their many other activities. They would, nevertheless, be prepared to implement special measures in a pandemic emergency. School nurses said that they were also too busy to promote safe hygiene routinely, but did sometimes do so during special health events. Nurses did, however, feel that infection control in the event of a pandemic would be their remit, and wished to have better guidance as to what to do in such a case. Children were well informed about the germ rationale for hygienic behaviour, but this did not translate into actual practice. Fun, hands-on activities and commercials using disgust approaches were highly appreciated by the children.

The study findings are limited by the small number of schools involved. We were not able to randomly select schools and classes within schools. Head teachers avoided selecting classes where low discipline was expected. Although we attempted to cover a broad range of socio-economic and ethnic backgrounds, the schools may not be representative as far as their interest in hygiene is concerned. However, despite large socio-economic and ethnic differences between the study schools, we found little variation amongst them as concerns the study findings.

### Implications for pandemic influenza preparedness

Schools are social environments in which children learn new behaviours both directly and vicariously. Teachers have an opportunity to directly control the behaviour of the children in class, and the teachers in our study did so by maintaining a limited number of activities, such as hand-washing before lunch and encouragement to use a tissue when sneezing. Infection control for the sake of public health did not appear to motivate teachers to encourage personal hygiene in children, but this could change during an influenza pandemic.

The study showed that teachers are able and willing to implement special measures to promote safe hygiene in the event of a pandemic. Whilst hand-washing with soap in existing washrooms was impractical and took too much time, teachers were able to maintain the use of gels 4 times a day over several days. Most of them thought that in the case of a severe public health threat, such measures could be maintained over several weeks.

The behaviour trials showed that regular classroom hand hygiene is greatly facilitated by using rinse-free hand sanitisers. Unlike Dyer [13] and White [14], we found little evidence suggesting that conventional alcohol-containing hand gels are not suitable and acceptable for children even when used over several weeks. It is questionable whether alcohol sanitisers are more damaging to the skin than soap and water [15]. Several teachers believed that using rinse-free sanitisers instead of soap may interfere with the educational effect of hygiene education. This may suggest that outside a pandemic infrequent scheduled hand hygiene occasions with soap and water could be more appropriate than frequent application of hand sanitisers. Also, it is not clear whether it is logistically feasible to provide all schools with an adequate supply of gels during a pandemic. The behaviour trials were well, at times even enthusiastically received by children and teachers, although this enthusiasm might have waned over a longer time period. Possibly, the behaviour trials themselves may have led to positive changes in children's and teachers' attitudes towards hygiene, but we did not collect long term data to confirm this.

Under the exceptional circumstances of a pandemic it appears feasible and acceptable to enforce intensive hygiene measures in children, especially since motivation in teachers and perhaps also children is likely to be much higher. Future research on hygiene interventions in schools could focus on practical and logistical aspects, for example identifying key times at which hand hygiene may potentially achieve the highest impact. School nurses, who may during a pandemic contribute to implementing these measures, felt that they needed more guidance. Such guidance could be delivered using a tested, practical motivational child-oriented toolkit.

### Implications for general infection control activities in schools

On the whole, the appropriateness of an intensive hand hygiene regimen outside a pandemic situation may be questionable. Given the barriers and constraints to extensive activities and the motivations of key stakeholders, it may be difficult to implement or even justify infection control measures targeting personal hygiene in school children that go beyond what is already happening, at least in the schools in our study.

We cannot confirm earlier studies suggesting that intensive hygiene interventions in schools, aiming at infection control outside a severe public health threat like an influenza pandemic [13,14,16], for example by using rinse-free hand-sanitisers [13,14], may be feasible and acceptable.

The available evidence does not necessarily support hand hygiene to be effective in reducing infections in schools and nurseries. Recent meta-analyses suggest a reduction of acute respiratory infections [17] and absenteeism [6]. However, the quality of most studies was described as inadequate [6,17]. The few high quality cluster randomised trials suggest, if at all, a very small effect of improving hand hygiene in schools and child care centres on infections [16,18].

## Conclusion

The scope of implementing intensive and regular hygiene activities in primary schools appears to be limited unless there is a major perceived public health threat like an influenza pandemic. This does not imply that there should be no hygiene education in schools at all. Schools, teachers and school nurses are motivated to contribute to hygiene education in addition to what children learn from their parents as long as the reality of the school environment is taken into account. With regard to pandemic influenza, hand hygiene and other hygiene measures in schools should remain a component in the range of public health measures during an influenza pandemic, unless future studies demonstrate the ineffectiveness of hand hygiene to specifically reduce the transmission of influenza.

## Competing interests

The hand hygiene products were provided free of charge by B.Braun Melsungen AG, Germany. The sponsor had no influence on the study and the manuscript.

## Authors' contributions

WPS and CW conceived the study, conducted the field work and drafted the manuscript, AB and VC contributed to developing the qualitative research components, and contributed to writing the manuscript. PM contributed to study design and writing the manuscript.

## Pre-publication history

The pre-publication history for this paper can be accessed here:


